# Theory and practice – a case study of coordination and ownership in the Bangladesh health SWAp

**DOI:** 10.1186/1478-4505-4-5

**Published:** 2006-05-16

**Authors:** Jesper Sundewall, Birger Carl Forsberg, Göran Tomson

**Affiliations:** 1 Karolinska Institutet, Department of Public Health Sciences, Division of International Health (IHCAR), SE-171 77 Stockholm, Sweden; 2 Medical Management Centre (MMC), Karolinska Institutet, Stockholm, Sweden

## Abstract

**Background:**

In the past decade the sector-wide approach (SWAp) model has been promoted by donors and adopted by governments in several countries. The purpose of this study is to look at how partners involved in the health SWAp in Bangladesh define ownership and coordination, in their daily work and to analyse the possible implications of these definitions.

**Methodology:**

The study object was a process of decision-making in the Government of Bangladesh in 2003. Information was collected through participant observations, interviews and document review.

**Results:**

During the study period the Government of Bangladesh decided to reverse a decision to unify the two wings of the Ministry of Health and Family Welfare. The decision led to disagreements with development partners, which had serious implications for cooperation between key actors in the Bangladesh health sector leading to deteriorated relationships and suspension of donor funds. The donor community in itself was also in disagreement which led to inconsistencies in the dialogue between the development partners and the Government of Bangladesh.

**Conclusion:**

The case shows that main actors in the Bangladesh health SWAp interpret ownership and coordination, fundamental aspects of SWAp, differently. As long as work ran smoothly, the different definitions did not create any problems, but when disagreements arose they became an obstacle. It is concluded that partners in development should devote more effort to their working relationships and that responsibilities within a SWAp need to be more clearly delineated.

## Background

Development assistance to health has been increasing during the last decade. At the same time, there has been a change in forms of assistance. Objectives have shifted from a more project-oriented approach towards control of specific diseases or strengthening of health systems. An increasing number of donors are also allocating a larger share of their development assistance to programmatic approaches or sector-wide approaches (SWAps) [1]. Since the SWAp model was introduced, we have noted a remarkable increase in the number of articles, reports and evaluations published about the model [2-6]. There is to date, however, limited documentation of how SWAps work in practice.

The SWAp model was introduced into development cooperation in the mid 1990s [7]. It has since then gained increasing popularity among development partners. More and more bilateral and multilateral development agencies are adopting and promoting SWAps as a preferred model of cooperation. It has also generally been accepted by a growing list of aid-receiving countries [8,9]. In Bangladesh, a sector-wide approach to health was introduced in 1998 with the Health and Population Sector Programme (HPSP). This meant a shift from project-based planning to sector-wide planning, management and financing [10].

There have been few attempts to define SWAp. Walford's [11] definition of SWAp, builds on an earlier definition which stated that a SWAp is when "all significant funding for the sector supports a single sector policy and expenditure program, under government leadership, adopting common approaches across the sector and progressing towards relying on Government procedures for all funds" [2,3,12]. Given this definition, Walford argues that a SWAp can be identified by the presence of the five elements listed in Table 1

**Table 1:** Elements present in a sector-wide approach. (Source: Walford 2003)

1. All significant funding agencies support a shared, sector-wide policy and strategy
2. A medium term expenditure framework or budget which supports this policy
3. Government leadership in a sustained partnership
4. Shared processes and approaches for implementing and managing the sector strategy and work program
5. Commitment to move to greater reliance on government financial management and accountability systems.

When reviewing publications on SWAp we found that few researchers have analyzed the sector-wide approach itself and addressed possible implications and outcomes of this new model of collaboration (For example: Peters and Chao [5], Walt et al [6], Buse and Walt [4] and Hill [13]). Even fewer have made attempts to analyze practical implications of SWAp (Jeppsson [14]and Buse [15] are two out of very few examples) and no study was found describing and analyzing, in detail, an activity or event within the SWAp process itself.

Sector-wide approaches has now been used in development for little more than a decade but is still evolving. It should be considered as a "work in progress" or a rhetoric open to debate [13]. Cassels and Janovsky [16] argue that the development of SWAps is a response to limitations of other forms of development assistance (e.g. project-based aid). SWAp has been argued for persuasively by claiming that it leads to increased health sector coordination and stronger national leadership and ownership [17].

### Coordination and Ownership in theory

Ownership and coordination can be seen as integral parts of a sector-wide approach. Hence, increased focus has been placed on this with the introduction of the SWAp model. As regards coordination, SWAp provides a broad framework within which all resources in the health sector are coordinated in a well-managed way, with recipients in the lead [6]. Program planning gets a joint donor-government perspective. Harrold and associates argued that increased coordination of partners and their activities should lead to less duplication of work and conflicting strategies and hence make more efficient use of resources [18]. For the purpose of this study, coordination suggest that partners in development should work to increase efficiency in development by collaborating in planning and implementation of activities, policy development and funding

Ownership on the other hand is commonly defined as a situation where the government has assumed leadership over the development process [11]. The term ownership is mainly focused on government ownership. Development partners, on the other hand give up ownership over projects in exchange for a voice in the broader sectoral development process [5]. In this paper, ownership is defined as when the government decides the direction and content of the development process after engaging in discussions with major stakeholders, including development partners.

Previous research has shown that what a sector-wide approach is, and how coordination and ownership is defined is not particularly clear [19]. The definitions of SWAp tend to vary both between levels, from model to actual implementation, but also between countries and between actors in countries where SWAp is being implemented. The purpose of this study is therefore to look at how partners involved in the SWAp in Bangladesh define ownership and coordination, fundamental aspects of the sector-wide approach model, in their daily work and to analyse the possible implications of those definitions.

## Methods

Data and information for this case study were collected in February/March 2003. The main method for data collection was participant observations [20]. We sat in on two HPSO Steering Committee and two Donor Consortium meetings as well as one extra meeting between the Ministry of Health and the development partners. The Annual Program Review Policy Dialogue (a meeting where representatives from the government, development partners and other major stakeholders in the health sector review the progress of the last year of the sector program) meeting in February 2003 was also observed. Furthermore, we conducted 16 semi-structured interviews with government officials and development partners who attended the abovementioned meetings. Respondents included high-ranking officials from different departments of the Ministry of Health and Family Welfare and representatives from all major (in terms of financial contribution) donors active in the health sector.

Observations were used as they allow for a conscious and systematic sharing of the interests and affects of a group [21]. The meetings attended were selected because they constitute the main consultative and coordinating meetings for stakeholders in the Bangladesh health SWAp. During the observations we studied how development partners and government representatives apply the ideas of coordination and ownership in their daily work and how they interpret these terms under different circumstances.

The interviews were used as a source for background information on the decision-making process that was being investigated. It was also used as follow-up and verification of what was observed during the meetings. The interviews were semi-structured in character and revolved mostly around a general discussion about the sector-wide approach model and how ownership and coordination can be defined and achieved.

Research papers and consultancy reports were also reviewed. These were obtained through *Google *and *Pubmed *searches using combinations of the keywords:*SWAp, sector-wide approach, ownership, coordination *and *Bangladesh*. Finally, official documents and reports concerning the HPSP produced by the Government of Bangladesh were reviewed. These documents were mainly provided by the interviewees. The review of articles and consultancy reports provided information on how coordination and ownership has been defined in the SWAp model. Documents concerning the HPSP on the other hand showed how ownership and coordination had been defined in the context of the Bangladesh SWAp specifically. The definitions of ownership and coordination form the basis for an analysis of how the concepts were translated by individual actors and applied in practice in a specific decision-making process in Bangladesh.

## Background to the case-study

### The Health and Population Sector Program (HPSP) in Bangladesh

The population in Bangladesh was at very high growth levels in the 1970s and 1980s. In spite of increasing efforts, it stubbornly refused to come down [22]. The urgent need for population control led to donor demand on the Government of Bangladesh to create a separate directorate within the Ministry of Health and Family Welfare (Ministry of Health) responsible for all family planning activities [23]. During the four health and population programs that were undertaken following independence in 1969, Bangladesh experienced a rapid growth in both number of donors as well as the amount of money available. The large aid presence resulted in a fragmented sector with little coordination and a large number of projects. The Fourth Health and Population Program alone accounted for around 75 different projects [24]. This was partly due to the fact that there was no mechanism established for coordination of donors [25]. During the 1990s, it was recognized that increased coordination and comprehensive reform of civil service was needed, which led to emphasis being placed on developing a sector-wide development program that would initiate reforms and increase efficiency. [23]

After reviewing and evaluating the Fourth Population and Health Project in Bangladesh, it was concluded that the focus on carrying out activities in the form of projects was inefficient [23]. The Health and Population Sector Program (HPSP), which commenced in 1998, therefore marked a change in the way health sector development was designed in Bangladesh. [26]. In the HPSP, a model of sector-wide management was adopted to plan the health and population sectors jointly.

With the HPSP major structural changes were introduced to strengthen the health system [27]. Up until the inception of the HPSP, there had been different structures for management and delivery of family planning services and regular health services. There had also, at every level, been very little collaboration between the two. In the design of the HPSP it was agreed that stronger integration in service delivery was needed in order to increase efficiency. One reform, which was clearly stated in the Memorandum of Understanding was therefore that there should be *"Unification of service delivery at thana level *(administrative level below districts)*and below" *[28].

The HPSP was designed and initiated during the reign of the Awami League party. It was the networking of pro-reform donors that had brought the reform agenda onto the political stage [25]. With the change of government in 2001, however, came a window of opportunity for critics of reforms under the HPSP. The planned unification process, in particular, was effectively stalled [10]. One reason for the strong opposition and criticism against the HPSP was that it was considered a product agreed upon only between development partners and the Prime minister at the time, Awami League's Sheikh Hasina.

**Figure 1:**
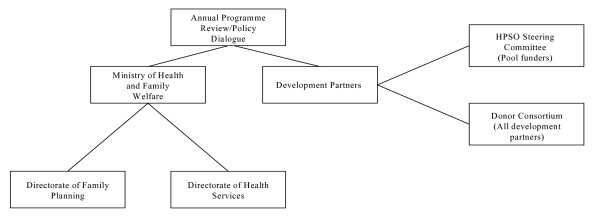
Overview of main institutions and coordinating bodies in the HPSP in Bangladesh.

With the HPSP, increased coordination of resources and activities undertaken by different partners was expected. Hence, special structures were created in Bangladesh for coordination of all stakeholders in health, including development partners. Figure 1 below provides a simple overview of the HPSP institutions and structures. The HPSO steering committee and the Donor Consortium are both structures for coordination of development partners. The HPSO Steering Committee is open only to those development partners that channel their funds into a common pool through which the overall health sector development program is supported. The Consortium is open to all development partners in the health sector. The Annual Program Review Policy Dialogue is mainly a meeting for the Ministry of Health and the development partners but is also attended by other stakeholders in the Health Sector.

## Results

### The issue of unification – ownership and coordination in practice

#### Background

The integration or "unification" of family planning and health services as described earlier was initiated early on in the HPSP. Steps were first taken to unify at sub-district level. The central level was to be unified last. All employees were to be put under one command. For instance, at district level, this meant that family welfare workers were to be put under the authority of the district medical officer, a person that they did not relate to in the prevailing set-up.

The unification met a lot of resistance within the Ministry of Health, especially from employees under the Directorate of Family Planning. This resistance made the reform move slowly. Development partners were pushing for the unification to be implemented but the Ministry of Health was struggling to overcome internal resistance.

In 2002, the Ministry of Health decided to initiate two independent studies of the unification issue in order to decide how to carry the process forward; one study by an independent team of consultants (the Independent Technical Team – ITT) and one study commissioned by the Independent Monitoring and Evaluation Division (IMED). The Ministry of Health had said that once the studies were presented, they would consult development partners before taking any decision on how to proceed with the issue of unification. The studies were presented in the beginning of 2003.

#### Reversing the reform

At the Annual Program Review Policy Dialogue in February 2003 the Ministry of Health announced that there would be no unification of the two wings, neither during the remainder of the Health and Population Sector Program, nor during the upcoming Health, Nutrition and Population Sector Program (HNPSP). Health and family planning services would also in the future be delivered separately through the existing, de-unified, structure.

Development partners were taken by surprise by this message and some of them immediately raised a number of concerns and objections. Their main concern was that this decision was not taken in collaboration with the development partners and that it was taken contrary to the recommendations given by the ITT study, which endorsed unification [29]. Since the decision was the complete opposite of the recommendations of the report, development partners asked the Ministry of Health to clarify the basis for their decision. The Ministry of Health referred to the IMED study which suggested that the de-unified structure should be preserved. Also, representatives from the family planning wing of the Ministry of Health stated that their services had not functioned well in the instances where they had been unified with the health services. According to the Independent Technical Report, however, the IMED study generally also endorsed unification [29]. Some development partners claimed that the conclusions of the IMED report had been changed from when it was circulated in draft form until it was final. There was no further clarification given from the government during the policy dialogue.

#### Development partners' reactions

Two heads of mission from large development partners made statements in the closing session of the policy dialogue. These statements showed large differences in the opinion about the health sector cooperation in Bangladesh. The first one indicated that he considered the HPSP a failure by saying "*We have invested millions of dollars, and uncountable hours during the Health and Population Sector Program and what results can we show? Not much. Then why should development partners continue to invest in the upcoming health sector development program?" *The second head of mission was more positive and reaffirmed his agency's commitment towards working with the Government of Bangladesh. He was pleased to see that the government had taken the lead in developing a conceptual framework for the next health sector program, which indicated strong government ownership over the process.

During the week after the policy dialogue, two meetings between development partners were scheduled; The Health and Population Support Office Steering Committee and the Consortium meeting. The meetings came to be completely dominated by the Ministry of Health's decision not to unify the two structures. At the Steering Committee meeting, the discussion from the policy dialogue continued.

One group of development partners considered the government to be in material breach with the agreement their institutions had with the government. The same partners also stressed the importance of taking action and showing the government that breaking an agreement has consequences. *"How credible is the government if they break agreements at will?" *one development partner asked. They suggested that development partners should cancel their funding to the health sector for the remainder of the program, or at least until the Ministry of Health had provided clarification on the unification issue. Another group of development partners did not consider the government's decision as a breach of agreement, nor did they want to take rapid action. *"We should not moralize too much. Changing commitments and priorities we see everyday in our own countries as well"*.

The discussion continued at the consortium meeting a couple of days later. Also in the consortium, it was clear that development partners had different opinions. The unification issue ignited the discussion but as it went along, it became more and more apparent that there were many other concerns among the development partners. It was even said that the current situation might make some development partners reconsider their commitment to the next program. Eventually, agreement was reached to draft a letter to the Ministry of Health. It was decided that the letter should not focus on the unification issue, but on the need for a good partnership and an open dialogue. The letter would also ask for clarification regarding how the Ministry of Health planned to reach the benefits that unification had been expected to generate, in its current plan.

In response to the request made by the development partners, the Secretary of the Ministry of Health called for a meeting with them. The Secretary stated that the meeting was to be seen as a start of a more extensive dialogue regarding the unification issue between the development partners and the government. A working paper that provided justification and rationale for the decision not to unify was distributed. The paper also presented how the Ministry of Health planned to achieve the benefits that were expected through a unified structure. The Secretary explained that the basis for the decision to de-unify was mainly political and was taken by the Prime Minister after a briefing from the Health Minister. He claimed that the political situation did not, at the moment, provide possibilities for unifying the two structures within the Ministry of Health. Most development partners were not convinced by the arguments presented but they all agreed that the meeting was a step towards improving the dialogue and relations.

A week or so later there was an extra Consortium meeting. The meeting had been called to discuss how development partners were considering their involvement in the upcoming health sector development program. Focus had been shifted from dealing specifically with the unification issue to a more general review of development partner's involvement in the health sector. A few development partners made it clear that, because of the deteriorating relationship between the Ministry of Health and the development partners, they were considering pulling out of the health sector and not fund the next program. The mistrust was not based solely on the decision regarding de-unification, but on other actions taken, or not taken, by the Ministry of Health as well. Other development partners however made it clear that they would not cancel their funding or reconsider their commitment.

On April 30 2003 however, the World Bank and those agencies that provided budgetary support ("pooled funding") decided to partially suspend their contribution to the health sector, an amount potentially as high as US dollar 65 millions [30]. According to the World Bank, the reason for the suspension was mainly the fact that the Ministry of Health's decision not to unify the two structures had been taken prior to consulting with development partners. The World Bank also stressed that the decision not to unify was merely one of several agreed reforms that had not been undertaken. The World Bank and its co-financiers promised to resume the credit as soon as the government presented an alternative reform agenda.

In response, the Health Minister said that the suspension would not hurt the government and that the IDA and the other development partners had not made any significant contribution to the health sector over the last five years. He also stressed that the government would continue to go by their needs and not abide by dictates from the IDA.

#### Outcome

The suspension of the credit was lifted in July 2003 after the Ministry of Health had presented a comprehensive plan to carry forward reforms to achieve the intended objectives of the HPSP. Slowly, trust was being restored between involved partners. The Ministry of Health however stood firm that there would not be any unification of the family planning and health wings.

The sector-wide approach was severely shaken by the events in Bangladesh. The issue of ownership was questioned and aspects of coordination disputed. Still, however, commitment to the sector-wide approach from partners in development stands strong. In recent policy documents the allegiance of all parties to move further into a sector-wide approach is clearly stated [31,32].

## Discussion

In this study we found that partners in the Bangladesh health SWAp define coordination and ownership differently. This is not very surprising. Jönsson has showed that international organisations, such as the WHO, facilitate the spread of new ideas and specific policies [33]. The SWAp model and with it the ideas of coordination and ownership can be seen as one such idea. These ideas, however, must be translated to a local context and accepted among stakeholders at country level. The sector-wide approach in Bangladesh has only been in effect for a few years which could perhaps explain the wide range of definitions articulated by different stakeholders.

### Coordination

Coordination lies at the heart of a SWAp. Instead of planning specific projects, partners in a SWAp agree on how resources are spent on common priorities [5]. A requirement for success is sufficient commitment to shared goals from both government and development partners [7]. In Bangladesh, forums for coordination of both development partners and the government have been in place for a number of years. The HPSP was also agreed upon by all partners but it did, however, not have a shared vision nor did it have jointly agreed upon operational definition of SWAp [34]. Hence, the disagreement can probably not be explained by lack of coordination. It was rather an argument concerning what should be coordinated and how.

According to Buse and Gwin [35], a consortium of development partners improves coordination by providing a venue for consultation, consensus building, optimizations of the comparative advantage of each contributor, and streamlining of interactions between donors and government. It is also known that personal relationships are important as individuals matter in the coordination process. No matter whether it is top politicians, consultants, project managers, within or outside the government, they all affect relationships within the policy environment in a myriad of ways [6]. In this case, the disagreements between development partners could be a result of too much focus on coordination of activities and resources with the government and too little focus on consensus building within the development partners' group. The lack of consensus could in turn possibly be explained by the lack of a common definition of ownership and by the deteriorated personal relationships between some partners.

### Ownership

With regard to ownership, the Health Minister of Bangladesh addressed the development partners at the Policy Dialogue, and said *"when you campaigned for the SWAp, you offered us the driver's seat"*. Other representatives from the government also argued that the Ministry of Health in a SWAp has the right to, and should, take whatever decision they deem necessary for improving the health sector. The Ministry of Health cannot let the development partners dictate the rules. Development partners on the other hand argued that there was no clear basis for the reversal of the unification [36]. Furthermore they stated that the Ministry of Health, of course, has every right to take sovereign decisions, but must also be prepared to face the consequences of them. From this discussion, it is evident that the notion of "offering the government the driver's seat" can be interpreted very differently.

Cassels and Janovsky [16] argued that SWAp is a model for increasing national ownership while still allowing for continued engagement from donors. The role of donors has changed from selecting which projects to finance to having a seat at the policy table [5]. In the literature however, little is said about the roles of different partners in a situation where there is a disagreement. In the case of Bangladesh, the government decided to go against the will of large development partners. Some donors saw this as a sign of true government ownership while others considered it a breach of partnership.

When development partners contemplated how to respond, it became clear that they had different views on how to best do so. While some thought that tough remedies should be taken against the government, others were more concerned about focusing on restoring the lack of confidence between the development partners and the government. While a couple of donors were considering pulling out of the health sector, others were not considering this as an alternative. There was consensus among all partners that they wanted to work with a sector-wide approach but there were many views of what this meant in reality. One lead development partner argued that *"if channelling money through the government is not the best way of achieving health outcomes, then we will consider other options"*. Other partners felt that the foundation of a sector-wide approach lies in working through the government and were not considering other alternatives.

### Limitations of the study

In this study we are accounting for an event as it happened. Observations provide a good tool for describing the complexity of a large scale development programme involving the government and several development agencies. One limitation, however, of participant observations as research method is that the interpretations of events, its reasons and implications, are made by the researchers. For this reason, follow-up interviews were made with most partners attending the observed meetings to complement the information amassed through the participant observations. Another limitation is that they study provides a snapshot of one period in time, for a controversy that was dealt with over a longer time-period. This fact limits the extent to which the analysis can account for effects of staff turnover among development partners and ongoing discussions in different factions of the Ministry of Health on perceptions of the problem and ability to find a solution.

## Conclusion

The SWAp model has spread rapidly in development cooperation. It has been applied and implemented in different contexts, under one label, but with different content. As we have seen in this study, definitions of ownership vary between partners but also between situations. It therefore seems that in Bangladesh, the notions of ownership and coordination have been accepted, but they have neither been discussed nor formalized. As long as work within the SWAp ran smoothly, these different definitions did not constitute a problem. When disagreements arose, however, the partners' different definitions of ownership and coordination became an obstacle. Everybody involved was supportive of the notions of ownership and coordination, but they had different views on what it actually meant. Hence we suggest that partners in development need to devote more attention to managing their working relationships. Furthermore we advise that roles and responsibilities within a sector-wide approach should be clearly delineated and that there should be appropriate mechanisms in place to handle potential disagreements between key stakeholders.

## Competing interests

The author(s) declare that they have no competing interests.

### Authors' contributions

JS designed and carried out the study and participated in writing the manuscript. BF participated in the analysis and interpretations of the findings and assisted in writing the manuscript. GT participated in writing the manuscript. All authors have read and approved the final version of the manuscript
